# Design of highly sensitive refractive index biosensor with cobalt-glass materials for detection of glucose concentration in blood

**DOI:** 10.1038/s41598-025-13498-y

**Published:** 2025-07-30

**Authors:** Trupti Kamani, Shobhit K. Patel, Sana Ben Khalifa, Saleh Chebaane, Om Prakash Kumar, Ishwar Bhiradi, Ammar Armghan

**Affiliations:** 1 Department of Physics, Marwadi University, Rajkot, 360003 India; 2 Department of Computer Engineering, Marwadi University, Rajkot, 360003 India; 3 Department of Physics, College of Science, Qassim University, P.O.Box 6644, Buraydah Almolaydah, 51452 Saudi Arabia; 4 Department of physics, College of Science, University of Ha’il, P.O.Box 2440, Ha’il, Saudi Arabia; 5 Department of Electronics and Communication Engineering, Manipal Institute of Technology, Manipal Academy of Higher Education, Manipal, 576104 India; 6 Department of Mechatronics, Manipal Institute of Technology, Manipal Academy of Higher Education, Manipal, 576104 India; 7 Department of Electrical Engineering, College of Engineering, Jouf University, Sakaka, 72388 Saudi Arabia

**Keywords:** Refractive index biosensor, Cobalt compounds, Biosensing techniques, Biomedical applications, Glucose concentration, High sensitivity, Parametric analysis, SDG 3 (Good health and Well-Being), SDG 9 (Industry, innovation and infrastructure), SDG 12 (Responsible consumption and production), Optical materials and structures, Optical techniques, Biomarkers, Endocrinology, Nanoscience and technology, Optics and photonics

## Abstract

Diabetes represents a conceivably fatal condition that occurs because of elevated blood glucose levels. The surface plasmon resonance (SPR) biosensor has emerged as currently the most sophisticated method for determining glucose levels. The blood glucose biosensor performance is determined by the refraction coefficient of the substance containing glucose in the form of molecules. In this study, we have offered Hollow-Ring Cubic-Structured Refractive Index Biosensor (HRCSRIB) for monitoring glucose levels in blood from normal concentrations of 165 to 180 (mg/dL) in compared to diabetic glucose concentrations of 182, 205, 220, 240, 255, 289, 255 (mg/dL). This biosensor detects variations in the refractive index of urine, which correlate with changes in blood glucose concentration. The presented biosensing technology uses cobalt compounds and their unique structure to analyze blood glucose levels by measuring the urine refractive value. The study of various parameters associated with geometrical shape dimensions has been also represented. This offered biosensor has an exceptional sensitivity with 1750 nm/RIU, and a detection limit of 0.000410. The exceptional value of the quality factor, as well as the figure of merit value, is 1670.07 nm/RIU for 182 mg/dL and 1241.13 for 205–289 mg/dL respectively. The best transmittance response of 6%, 9%, 4%, and 32% has been obtained. The aforementioned sensor’s distinctive characteristics facilitate its biomedical applications as an optical on-chip sensor that monitors level of glucose.

## Introduction

Photonic biosensors are found to continue to increase in demand across investigators and researchers while they explore exciting new possibilities in healthcare technology. Surface plasmon resonance, resonate mirror technique, interferometry, and fluorescent spectroscopy belong to the several optical approaches currently in use for biological detection purposes^1–3^. The use of surface plasmon resonance (SPR) is being thoroughly studied for biological sensors^4–7^ as well as providing numerous advantages, which include versatility in designing, a reduction in size, the multiplexing process of detecting information, and the ability to be tracked remotely^8,9^. SPR-based bio-sensor devices have since become commercially readily accessible and have already been effectively evaluated for a broad variety of applications within the discipline of detecting multiple biological molecules, which include protein, growth hormones, amino acids, genetic material (DNA), antigens, various enzymatic agents, medications, and the quality of food^10–12^. Furthermore, SPR-based biosensing techniques exhibits assured clearly advantageous features over traditional biosensors, thereby paving the path towards a label-free analysis of various analytical substances and immediate evaluation without the necessity to utilize complicated laboratory setups or radiological or fluorescent labels^13,14^.

Amongst the several different microscopic molecules that exist in the blood of individuals, measuring the amount of glucose serves as vital for assessing general health, particularly in those suffering from diabetes^15^. In accordance with the ADA (American Diabetes Association), someone who is in good health ought to maintain a postprandial glucose concentration in their blood not exceeding 180 mg/dL along with a preprandial blood glucose concentration which varies between 90 and 130 mg/dL^16^. According to the range of values outlined above, elevated levels of blood glucose give rise to diseases such as diabetes, and this has emerged as a significant illness that impacts modern sophisticated society. Additionally, reducing glucose content in the blood eventually leads to hyperglycemia which could influence the cerebral and the accurate development of brain cells. Consequently, it is now necessary to accurately measure blood glucose levels at regularly scheduled times for the purpose of keeping a healthy lifestyle. Being aware of the significance of an accurately measured amount of glucose, the researchers have therefore worked on precise glucose concentration monitoring in our study according to an SPR-based biosensor^17^.

Many different kinds of SPR-based biological sensor properties and biomedical applications have recently been studied and published^18–20^. Mojtaba Hosseinzadeh Sani et al. present a study of diabetes, cancer, blood components detection, and glucose level detection with the help of an optical biosensor based on resonance cavities in the 2-dimensional photonic crystal. It also has the ability to distinguish between cancerous patients and a normal individual during the 1.19760511 to 1.19760519 μm resonating wavelength region. The measured level of glucose presents in ordinary condition urinals around (0–15 mg/dL), as well as urinals containing 0.625, 1.25, 2.5, 5, together with 10 mg/dL of glucose, was measured and analyzed across a wavelength spectrum of 1.19760504 to 1.19760510 μm^21^. Md. Rajibur Rahaman Khan et al. propose a fiber optic biosensor with a wide dynamic range for the determination of the PH and glucose concentration. The Fabry-Perot optical interferometry technology is used as a basis for the presented biosensor’s working idea process. In this sensor, to develop 5 pH levels along with 3 blood glucose fiber-optic probing sensors, their membranes were then placed on 8 nanoparticles composed of gold deposited onto fiber-optic probing. This sensor achieves a sensitivity is 1.95 nm/PH and 3.25 nm/mM for PH and glucose concentration detection respectively with a 2.5% standard deviation^22^. Sandeep Kumar Chamoli et al. introduce a 1-D refractive index biosensor to detect blood concentration with barium flint glass array, gold material, and silicon nitride substrate. The optoelectronic sensitivity of the introduced geometry in glucose level monitoring is being determined by analyzing the impact of the environmental refraction index or concentration of glucose along with the 1500 nm/RIU quality factor value^23^. Hongdan Wan et al. demonstrate optofluidic microcapillary biosensors for the identification of low-concentration glucose with label-free detection. In this manuscript, optofluidic microcapillary has been employed to bio-chemically functionalize to determine a low glucose concentration of 2.78 mM. The sensitivity of 0.966 pm/mM with a quality factor of 1.3 × 10^6^ has been determined and the presented microcapillary has 23.36 nm/RIU sensitivity of refractive index. The study shows that results depend on the thickness of the microcapillary as well as the refractive index of a liquid core^24^. Arslan Asim et al. designed a surface plasmon resonance metasurface-based refractive index sensor for aqueous glucose levels. The sensor’s structure uses metallic material nano-cylinders and a metal-dielectric-metal arrangement for achieving almost uniform absorbency across the 1800–2200 nm band of near-infrared wavelengths. Using Finite Difference Time Domain (FDTD) modelling, the design was examined. The sensor’s maximum sensitivity along with the Figure of Merit (FOM) is around 500 nm/RIU and 11.82 RIU1, respectively, and it demonstrates a linear response^25^. Shahriar Mostufa et al. reported a graphene layered SPR-based biosensor for determining the glucose level in blood and urine employing a finite element method. This SPR-based sensor exhibiting an angular sensitivity of 200 deg/RIU was scientifically shown to identify a 6.1025 g/l rise in hemoglobin levels in the component of blood that results from a 0.001 refraction index increase^26^.

This work offers a Hollow-Ring Cubic-Structured Refractive Index Biosensor (HRCSRIB) for the detection of glucose levels in the blood samples of concentrations varying from 165 to 342 mg/dL in which up to 180 mg/dL is considered as a normal concentration in the blood samples^27^. The offered sensor precisely measures diversified amounts of glucose according to their refractive index value changes in the urine samples. The presently modeled HRCSRIB biosensor serves as label-free identification, a simple manufacturing process, quick response time, a satisfactory solution, as well as a broad detection area.

## Sensor geometry and mathematical model

### Biosensor design structure

**Fig. 1:**
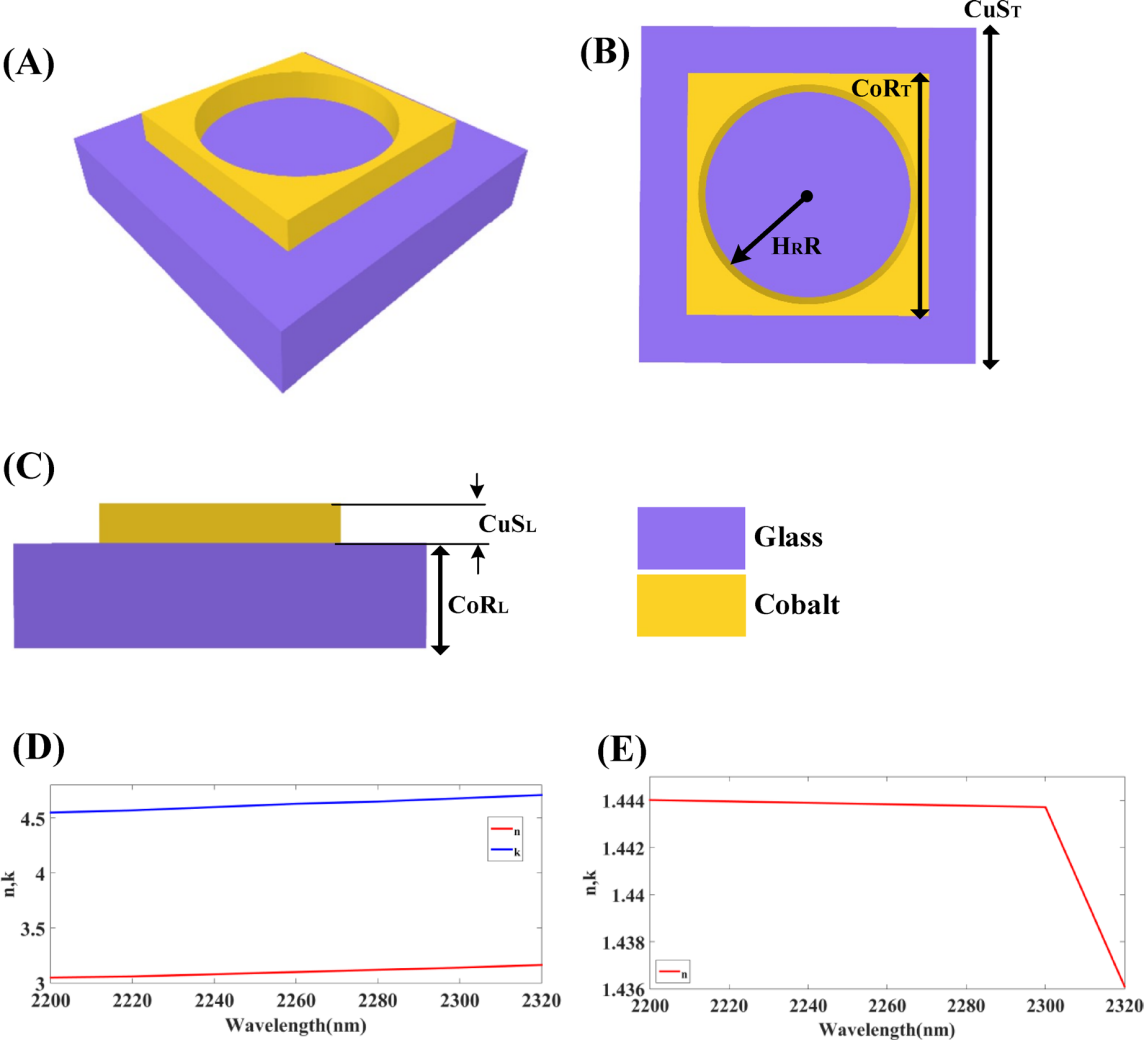
Graphic description of an offered Hollow-Ring Cubic-Structured Refractive Index Biosensor (HRCSRIB) **(A)** 3D scene of HRCSRIB **(B)** Top scene of HRCSRIB **(C)** Side scene of HRCSRIB **(D)** Refractive index, Extinction coefficient (n, k) versus wavelength plot of cobalt (Co) **(E)** Refractive index (n) versus wavelength plot of silicon dioxide (SiO_2_).

The following part of the biosensor gives an idea for designing a structure of a Hollow-Ring Cubic-Structured Refractive Index Biosensor (HRCSRIB) for glucose level identification. Here, glass is employed as a substrate material, and cobalt material is employed as a hollow ring cubic resonator. The combination of cobalt material together with the Hollow-Ring Cubic Structure design results in a synergistic platform that substantially optimizes the performance of the offered biosensor. Cobalt serves as an interesting material for biosensor design because of its chemical versatility, efficiency, affordability, and plasmonic characteristics. Cobalt has superior stability under biological conditions, including blood, primarily when used in its metallic and oxide forms. Its redox-active nature, which includes many states of oxidation such as Co²⁺ and Co³⁺, provides increased transmission of signals but needs careful control to prevent unintentional interactions with biomolecules. If unbound cobalt ions have not been neutralized, they may attach to plasma proteins and cause cellular damage, which may generate a barrier to biosensor biocompatibility. These risk factors can potentially be mitigated through the incorporation of cobalt into structured nanomaterials and providing protective surface coatings that protect cobalt from direct contact with blood components. From the perspective of sustainability, cobalt has distinct benefits over the noble metals because of its higher abundance as well as the minimized cost of the material. Here, in the context of the presented HRCSRIB biosensor, cobalt’s uses as a resonating material give enhanced plasmonic response and sensitivity. When combined with specially targeted glucose-binding substances, this HRCSRIB design makes it possible for efficient and precise determination while ensuring the structural stability in biological samples. Cobalt’s exceptional electrostatic properties consequence in more significant resonant dips across the SPR curve, which enhances determination precision and sensitivity. This metal’s affordable price provides it with a desirable substitute for more costly expensive metals such as silver and gold, thus enabling the development of highly effective and inexpensive biosensors. When it is paired with a hollow-ring cubic-structure, which exploits its distinctive shape to measure glucose levels in the blood by determining changes in the refractive value of samples of urine. The hollow-ring layout optimizes sensitivity towards refractive index variations by presenting a wider surface area for interactions with the analyte, which leads to more accurate detection. The idea behind it is a convenient and efficient way of diagnosis that not only improves the sensitiveness as well as precision of glucose detection but also offers non-invasive testing using samples of urine. By implementing these geometrical properties, the biosensor serves as an affordable and trustworthy choice for measuring blood glucose in healthcare applications. The optimum dimensions of the above proposed HRCSRIB refer to the cobalt-resonator length (C_O_R_L_) as 50 nm, and the cubic-shaped substrate length (CuS_L_) as 200 nm. The dimension of cobalt-resonator thickness (C_O_R_T_) is 660 nm, the cubic-shaped substrate thickness (CuS_T_) is 1200 nm, and the hollow-ring resonator radius (H_R_R) is 300 nm. Figure 1 showcased the graphic description of an offered Hollow-Ring Cubic-Structured Refractive Index Biosensor (HRCSRIB). Figure 1(A) showcased the 3D scene, Fig. 1(B) showcased a top scene, and Fig. 1(C) showcased a side scene of the HRCSRIB. In the context of refractive index, the offered HRCSRIB used cobalt material as a resonating element in the structural design, which exhibits distinct dispersion properties throughout the visible and near-infrared spectrums. Its refractive index (n) and coefficient of extinction (k) are not kept constant rather increase with wavelength, and it showcased in Figs. 1(D) and 1(E). Figure 1(D) plot shows the response of cobalt possessing *n* = 3.05 and k = 4.55 at a wavelength of 2200 nm. At wavelength λ = 2240 nm, the values are *n* = 3.08 and k = 4.6, and at λ = 2280 nm, the values are *n* = 3.12 and k = 4.65. As the wavelength increases in the near-infrared area up to λ = 2320 nm, the values increase to *n* = 3.16 and k = 4.71. This optical variance serves as an essential factor to generate significant plasmonic resonance and sharper spectrum responses, which directly lead to enhanced sensitivity and specific peak detection. In a similar way, the base material, silicon dioxide, showcases modest dispersion, primarily in the near infrared spectrum shown in Fig. 1(E). Figure 1(E) plot shows the response of base material (Silicon dioxide) possessing *n* = 1.44402 at a wavelength of 2200 nm. At wavelength λ = 2240 nm, the value of *n* = 1.44390, and at λ = 2280 nm, the value of *n* = 1.44378. As the wavelength increases up to λ = 2320 nm, the values decrease to *n* = 1.43611. Here, the extinction coefficient is zero over the range. By considering its wavelength-dependent refractive index variation instead of assuming it as a constant, we diminish simulation errors as well as enhance the spectrum response accuracy. Both the glucose level as well as the light wavelength have an impact on the sample layer’s refractive index, which corresponds to blood containing various glucose concentrations. Increased concentrations of glucose have been observed that can lead to a small but detectable increase in the refractive index, and biological transmission and scattering processes even further modify this change by wavelength. By incorporating all of these wavelength-sensitive optical behaviours into our computational structure, we enhance prediction accuracy in resonance shift, optimize biosensor operational metrics, and coordinate with the layout to actual operational conditions.

The numerical modelling setup of the offered Hollow-Ring Cubic-Structured Refractive Index Biosensor (HRCSRIB) has been simulated by employing COMSOL Multiphysics. A normal mesh has been implemented all over the domain of computation, which ensures an appropriate balance between computational efficiency and high spatial resolution. Periodic boundary conditions were implemented at the sides in order in order to accurately represent the sensor array’s periodic properties. Port boundary conditions had been applied to the upper and lower surfaces of the simulation domain to determine the incident and transmitted electromagnetic waves, respectively. These ports enabled the stimulation and detection of wave propagation inside the biosensor structure. The optical properties of cobalt and glass have been defined by wavelength-dependent diverse refractive indices, which allow accurate modelling of plasmonic and dielectric effects in the near-infrared region. The fabrication of an HRCSRIB biosensor for glucose detection is technically feasible using a combination of advanced nanofabrication techniques and methods. This unique hollow-ring cubic shape could have been fabricated with high accuracy and reliability using techniques like focused ion beam milling, template-based electrodeposition, or two-photon polymerization-based three-dimensional nanoprinting. These approaches are feasible for microfluidic integration, which makes the HRCSRIB suitable for use as on-chip biosensing devices. Cobalt-based nanomaterials could potentially be deposited or designed on their structure by utilizing sputtering, electrochemical deposition, or atomic layer deposition, which ensures homogeneous coating and multifunctional chemical properties of the surface for glucose-specific interactions. Due to its high compatibility and miniaturization, the offered HRCSRIB biosensor is suitable for in vivo application while detecting glucose concentrations. While the fabrication of the proposed hollow ring cubic biosensor for glucose detection poses some critical challenges that including achieving a nanoscale structural accuracy and the need to maintain the stability of cobalt material. The presented design effectively minimises these limitations by coordinating with modern nanofabrication techniques and implementing protective processing methods. Advanced lithography methods, such as electron beam and nanoimprint lithography, that facilitate high-resolution imprinting of complex cubic ring geometries, along with providing uniform plasmonic performance across devices. The presented HRCSRIB biosensor’s symmetrical and flexible structure improves its compatibility with versatile and reproducible fabrication, which allows for possible real-world application while addressing both sensitivity and manufacturability.

### Substantial parameters

The mathematical model of the offered Hollow-Ring Cubic-Structured Refractive Index Biosensor (HRCSRIB) includes some substantial parameters like quality factor, detection limit, signal noise ratio, sensitivity, sensor resolution, a figure of merit, detection range, etc. that can be introduced with the efficiency of the biosensor. Mathematical equations from eq. no. 1 to 7 of the HRCSRIB of substantial parameters are tabulated in Table 1. The functionality of the biosensor depends on some factors like the geometry design of the sensor, dimensions, materials used in a substrate, the material used in a resonator, and the type of the sensor.

**Table 1:** Substantial parameters of an offered Hollow-Ring Cubic-Structured refractive index biosensor (HRCSRIB)^28–30^.

Sr. No.	Substantial parameters	Equations
1.	Quality Factor	$\:Q=\frac{\lambda\:}{FWHM}$
2.	Detection Limit	$\:DL=\left(\frac{\varDelta\:n}{1.5}\right)\times\:{\left(\frac{FWHM}{\varDelta\:\lambda\:}\right)}^{1.25}$
3.	Signal Noise Ratio	$\:SNR=\frac{\varDelta\:\lambda\:}{FWHM}$
4.	Sensitivity	$\:S=\frac{\varDelta\:\lambda\:}{\varDelta\:n}$
5.	Sensor Resolution	$\:SR=S\:\times\:DL$
6.	Figure of Merit	$\:FOM=\frac{S}{FWHM}$
7.	Detection Range	$\:DR=\frac{\lambda\:}{\sqrt{FWHM}}$

## Results & discussions

Parametric dimensional assessments in biosensors include researching how different geometrical and material properties impact sensor functionality. The offered Hollow-Ring Cubic-Structured Refractive Index Biosensor assessments of parametric dimensions in the wavelength spectrum of 2260 nm to 2320 nm are shown in this section of results and discussions. This research involves gazing at possible effects of specifications like the thickness of the sensing layer, element spacing, sensing dimension of the elements, and shape.

**Fig. 2:**
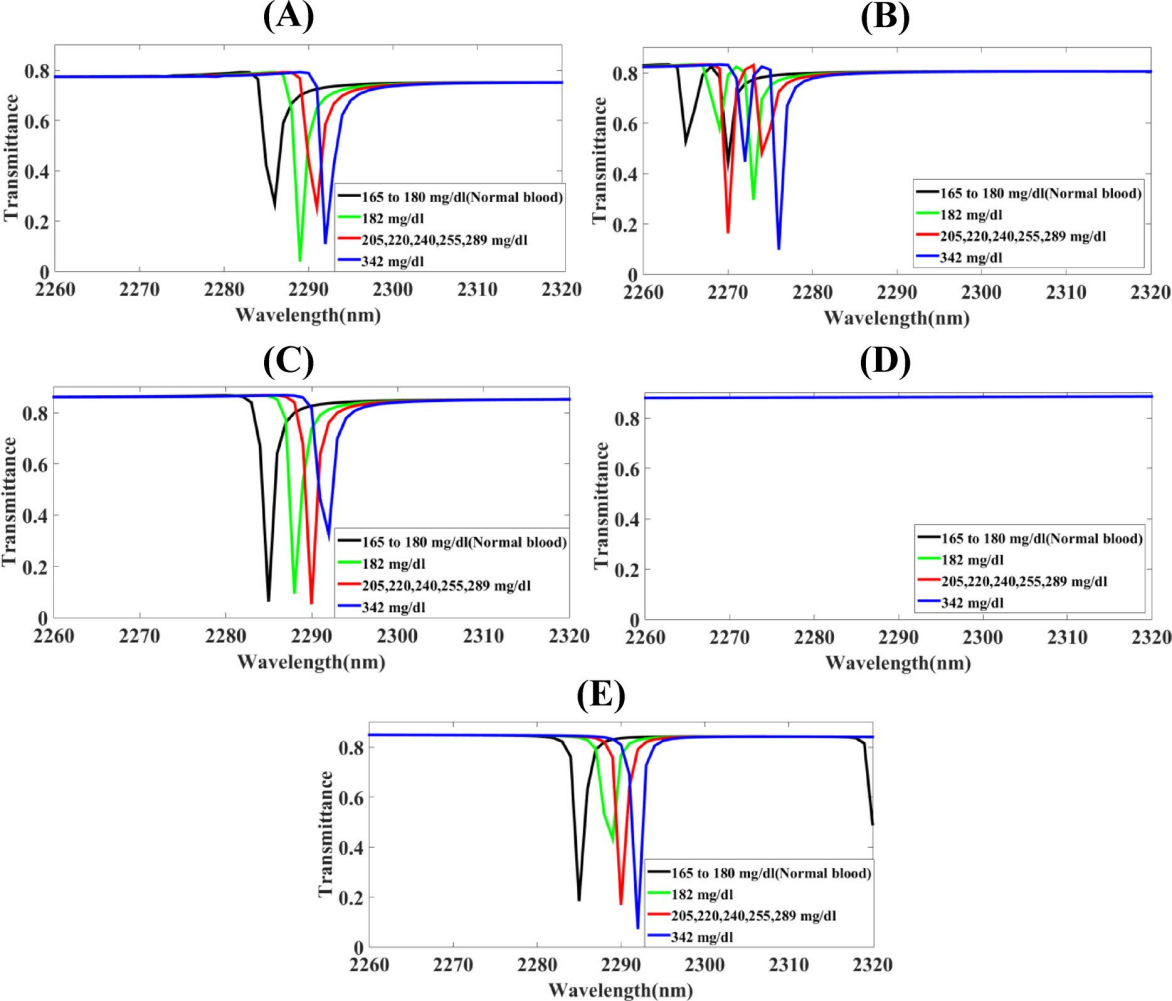
The screening of substantial parameters of the Hollow-Ring Cubic-Structured Refractive Index Biosensor (HRCSRIB) that has changed parameters (nm) and functions at a hollow-ring resonator radius in its wavelength range of operation of 2260 to 2320 nm **(A)** H_R_*R* = 260 **(B)** H_R_*R* = 280 **(C)** H_R_*R* = 300 **(D)** H_R_*R* = 320 **(E)** H_R_*R* = 340.

The screening of substantial parameters of the Hollow-Ring Cubic-Structured Refractive Index Biosensor (HRCSRIB) that has changed parameters (nm) and functions at a hollow-ring resonator radius in its wavelength range of operation of 2260 to 2320 nm is showcased in Fig. 2. Figure 2(A) showcases the screening of functions at a hollow-ring resonator radius H_R_*R* = 260 nm, as well as Fig. 2(B) showcases the screening of functions at a hollow-ring resonator radius H_R_*R* = 280 nm, where overlapping multiple peaks introduce a form of interruption that may affect accurate detection. On the contrary, Fig. 2(C) showcases the screening of functions at a hollow-ring resonator radius H_R_*R* = 300 nm. Moreover, Fig. 2(D) showcases the screening of functions at a hollow-ring resonator radius H_R_*R* = 320 nm with no response in detecting peaks, while Fig. 2(E) showcases the screening of functions at a hollow-ring resonator radius H_R_*R* = 340 nm. A thorough assessment using a parametric method has concluded that Fig. 2(C) shows a significant change in sensor response, and this relates to the placed HRCSRIB structure hollow-ring resonator radius H_R_R, becoming an exceptional sensitivity with 1750 nm/RIU for 342 mg/dL glucose level, 1666.66 nm/RIU for 205–289 mg/dL glucose level, and 1500 nm/RIU for 182 mg/dL glucose level. The exceptional value of the quality factor, as well as the figure of merit value, is 1670.07 nm/RIU of 182 mg/dL and 1241.13 for 205–289 mg/dL. The exceptional value of detection limit with the 0.000410 and best transmittance response of 6%, 9%, 4%, and 32% has been noted. Here, transmittance response suggests changes in dip sharpness, which corresponds to the surface plasmon resonance behaviour. This behaviour shows that electromagnetic fields strongly coupled with the conduction electrons at the metal dielectric interface. The different transmission ratios of the glucose detection peaks indicates that lower and sharper transmittance dips correspond to stronger plasmonic behaviour strong. The detection range of 1955.55 has been noted for 182 mg/dL concentration of glucose. Here, a lower response makes a difference from the normal one & that can be detected in an accurate manner. Figures 2(A) & 2(E) shows a higher transmission response with the values of 27%, 5%, 25%, 11%, and 19%, 44%, 17%, 8% for the normal concentration of glucose of 165–180 mg/dL, 182 mg/dL, 205–289 mg/dL, and 342 mg/dL respectively.

**Fig. 3:**
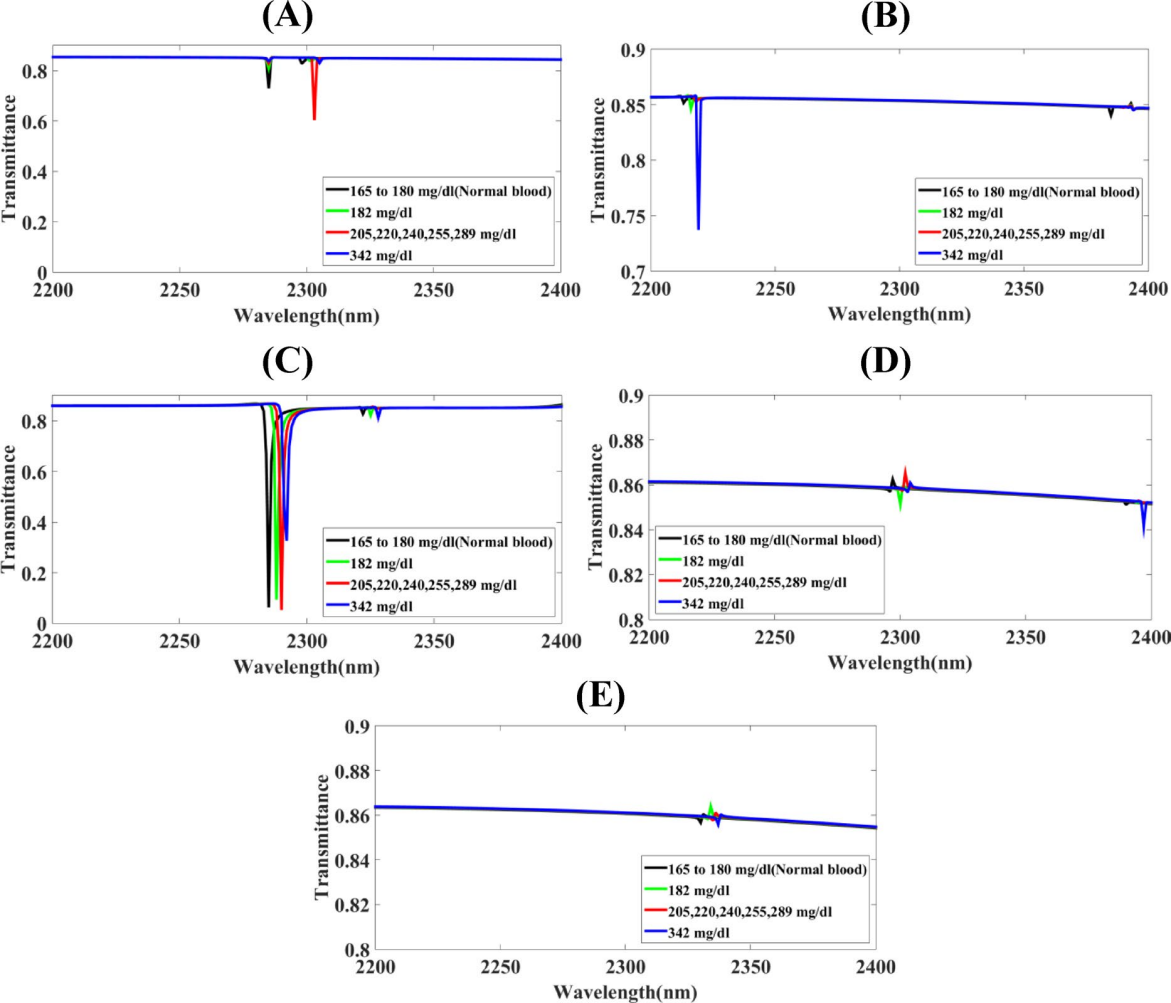
The screening of substantial parameters of the Hollow-Ring Cubic-Structured Refractive Index Biosensor (HRCSRIB) that has changed parameters (nm) and functions at a cubic-shaped substrate thickness in its wavelength range of operation of 2200 to 2400 nm **(A)** CuS_T_ = 1180 **(B)** CuS_T_ = 1190 **(C)** CuS_T_ = 1200 **(D)** CuS_T_ = 1210 **(E)** CuS_T_ = 1220.

The screening of substantial parameters of the Hollow-Ring Cubic-Structured Refractive Index Biosensor (HRCSRIB) that has changed parameters (nm) and functions at a cubic-shaped substrate thickness in its wavelength range of operation of 2200 to 2400 nm is showcased in Fig. 3. Figure 3(A) showcases the screening of functions at a cubic-shaped substrate thickness CuS_T_ = 1180 nm, as well as Fig. 3(B) showcases the screening of functions at a cubic-shaped substrate thickness CuS_T_ = 1190 nm. On the contrary, Fig. 3(C) showcases the screening of functions at a cubic-shaped substrate thickness CuS_T_ = 1200 nm. Moreover, Fig. 3(D) showcases the screening of functions at a cubic-shaped substrate thickness CuS_T_ = 1210 nm, while Fig. 3(E) showcases the screening of functions at a cubic-shaped substrate thickness CuS_T_ = 1220 nm. A thorough assessment using a parametric method has concluded that Fig. 3(C) shows a significant change in sensor response, and this relates to the placed HRCSRIB structure cubic-shaped substrate thickness CuS_T_, becoming an exceptional sensitivity with 1750 nm/RIU for 342 mg/dL glucose level, 1666.66 nm/RIU for 205–289 mg/dL glucose level, and 1500 nm/RIU for 182 mg/dL glucose level. Figures 3(A) shows a higher transmission response with the values of 83%, 84%, 61%, 83%, as well as Figs. 3(B) obtain very high response of transmission values of 85%, 84%, 85%, 74% for the normal concentration of glucose of 165–180 mg/dL, 182 mg/dL, 205–289 mg/dL, and 342 mg/dL respectively. Here, a higher response may create interruption while monitoring the concentration of glucose. Figures 3(D) & 3(E) showcase very high transmission rates around 86% with very nearness of the detection peaks.

**Fig. 4:**
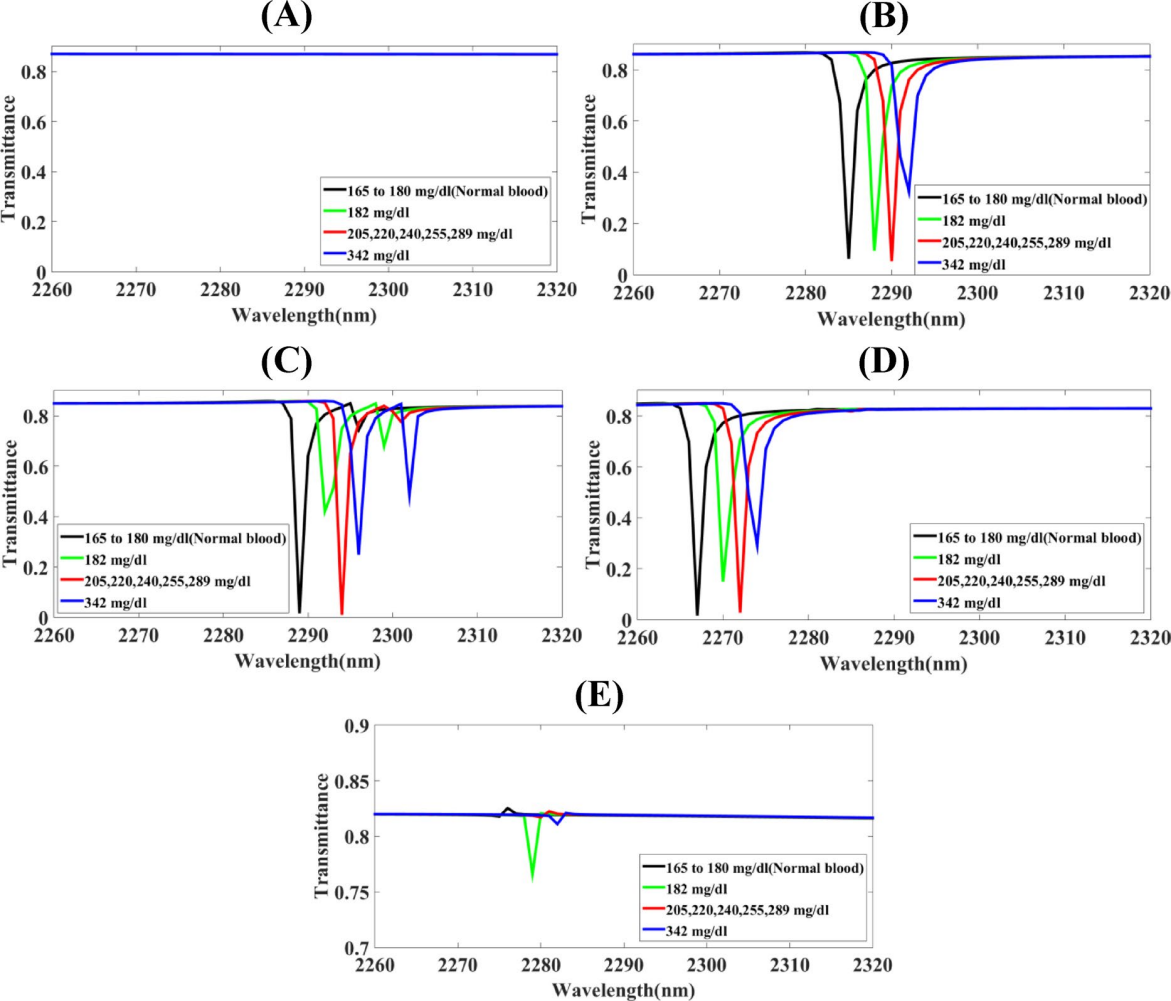
The screening of substantial parameters of the Hollow-Ring Cubic-Structured Refractive Index Biosensor (HRCSRIB) that has changed parameters (nm) and functions at cobalt (Co)-based resonator thickness in its wavelength range of operation of 2260 to 2320 nm **(A)** CoR_T_ = 650 **(B)** CoR_T_ = 660 **(C)** CoR_T_ = 670 **(D)** CoR_T_ = 680 **(E)** CoR_T_ = 690.

The screening of substantial parameters of the Hollow-Ring Cubic-Structured Refractive Index Biosensor (HRCSRIB) that has changed parameters (nm) and functions at cobalt (Co)-based resonator thickness in its wavelength range of operation of 2260 to 2320 nm is showcased in Fig. 4. Figure 4(A) showcases the screening of functions at cobalt (Co)-based resonator thickness CoR_T_ = 650 nm with a straight line (no peak detected at this dimension), as well as Fig. 4(B) showcases the screening of functions at cobalt (Co)-based resonator thickness CoR_T_ = 660 nm. On the contrary, Fig. 4(C) showcases the screening of functions at cobalt (Co)-based resonator thickness CoR_T_ = 670 nm. Moreover, Fig. 4(D) showcases the screening of functions at cobalt (Co)-based resonator thickness CoR_T_ = 680 nm, while Fig. 4(E) showcases the screening of functions at cobalt (Co)-based resonator thickness CoR_T_ = 690 nm. A thorough assessment using a parametric method has concluded that Fig. 4(B) shows a significant change in sensor response, and this relates to the placed HRCSRIB structure cobalt (Co)-based resonator thickness CoR_T_, becoming an exceptional sensitivity with 1750 nm/RIU for 342 mg/dL glucose level, 1666.66 nm/RIU for 205–289 mg/dL glucose level, and 1500 nm/RIU for 182 mg/dL glucose level. Figure 4(C) having multiple peaks getting overlapped which has one kind of interruption while monitoring. Figure 4(D) dimensions gives less sensitivity, less quality factor and less functioning ability than CoR_T_ = 660 nm, and Fig. 4(E) showcase transmission rates near by 80% at the 2278 nm wavelength range.

**Fig. 5:**
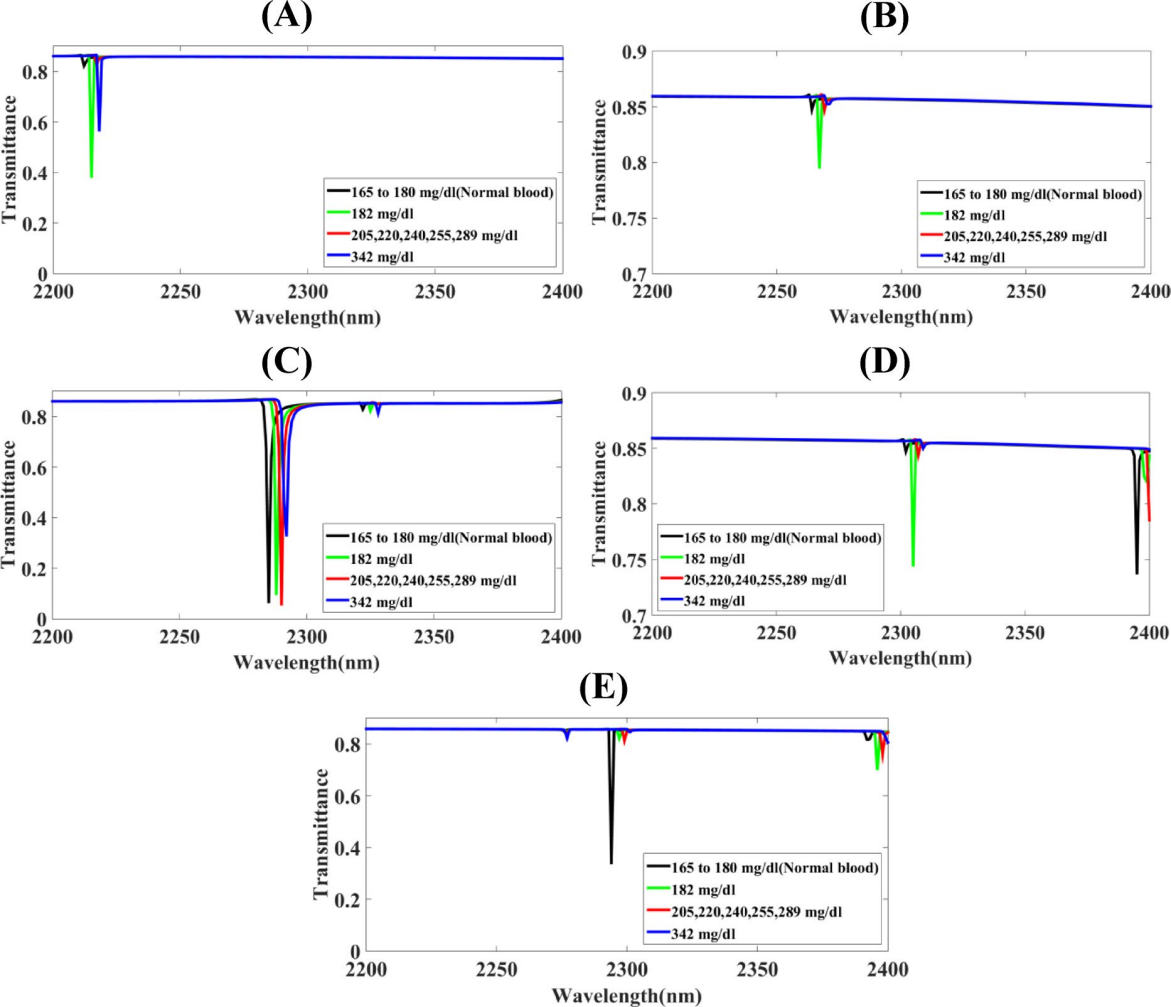
The screening of substantial parameters of the Hollow-Ring Cubic-Structured Refractive Index Biosensor (HRCSRIB) that has changed parameters (nm) and functions at a cubic-shaped substrate length in its wavelength range of operation of 2200 to 2400 nm **(A)** CuS_L_ = 160 **(B)** CuS_L_ = 180 **(C)** CuS_L_ = 200 **(D)** CuS_L_ = 220 **(E)** CuS_L_ = 240.

The screening of substantial parameters of the Hollow-Ring Cubic-Structured Refractive Index Biosensor (HRCSRIB) that has changed parameters (nm) and functions at a cubic-shaped substrate length is showcased in Fig. 5. Figure 5(A) showcases the screening of functions at a cubic-shaped substrate length CuS_L_ = 160 nm, as well as Fig. 5(B) showcases the screening of functions at a cubic-shaped substrate length CuS_L_ = 180 nm. On the contrary, Fig. 5(C) showcases the screening of functions at a cubic-shaped substrate length CuS_L_ = 200 nm. Moreover, Fig. 5(D) showcases the screening of functions at a cubic-shaped substrate length CuS_L_ = 220 nm, while Fig. 5(E) showcases the screening of functions at a cubic-shaped substrate length CuS_L_ = 240 nm. A thorough assessment using a parametric method has concluded that Fig. 5(C) shows a significant change in sensor response, and this relates to the placed HRCSRIB structure a cubic-shaped substrate length CuS_L_, becoming an exceptional sensitivity with 1750 nm/RIU for 342 mg/dL glucose level, 1666.66 nm/RIU for 205–289 mg/dL glucose level, and 1500 nm/RIU for 182 mg/dL glucose level. The higher responses of transmission with the values of 82%, 38%, 57%, 83% obtained in Fig. 5(A), 85%, 80%, 84%, 85% obtained in Fig. 5(B), 85%, 75%, 84%, 85% obtained in Fig. 5(D), and 34%, 83%, 82%, 85% obtained in Fig. 5(E).

**Fig. 6:**
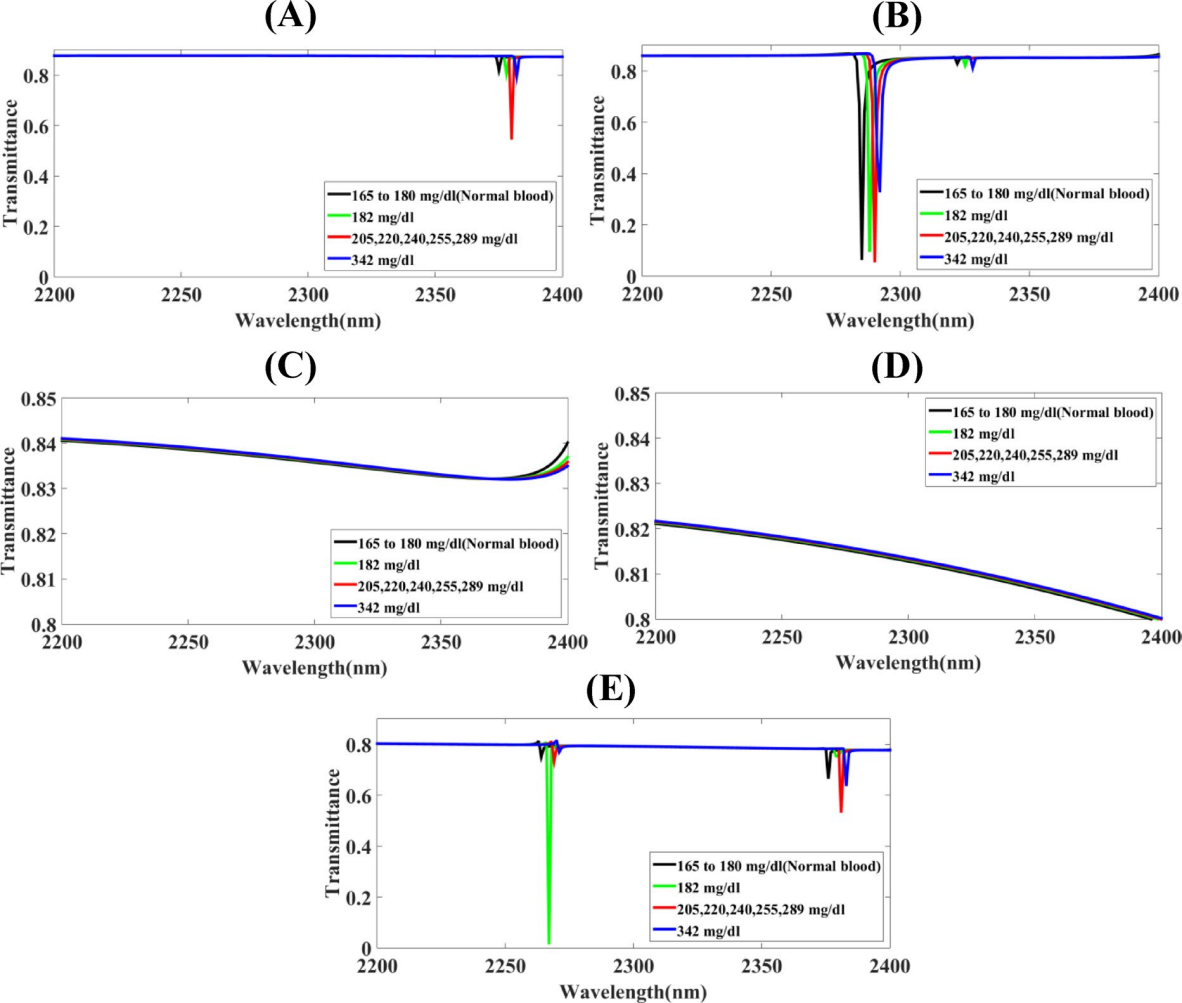
The screening of substantial parameters of the Hollow-Ring Cubic-Structured Refractive Index Biosensor (HRCSRIB) that has changed parameters (nm) and functions at cobalt (Co)-based resonator length in its wavelength range of operation of 2200 to 2400 nm **(A)** CoR_L_ = 40 **(B)** CoR_L_ = 50 **(C)** CoR_L_ = 60 **(D)** CoR_L_ = 70 **(E)** CoR_L_ = 80.

The screening of substantial parameters of the Hollow-Ring Cubic-Structured Refractive Index Biosensor (HRCSRIB) that has changed parameters (nm) and functions at cobalt (Co)-based resonator length is showcased in Fig. 6. Figure 6(A) showcases the screening of functions at cobalt (Co)-based resonator length CoR_L_ = 40 nm, as well as Fig. 6(B) showcases the screening of functions at cobalt (Co)-based resonator length CoR_L_ = 50 nm. On the contrary, Fig. 6(C) showcases the screening of functions at cobalt (Co)-based resonator length CoR_L_ = 60 nm. Moreover, Fig. 6(D) showcases the screening of functions at cobalt (Co)-based resonator length CoR_L_ = 70 nm, while Fig. 6(E) showcases the screening of functions at cobalt (Co)-based resonator length CoR_L_ = 80 nm. A thorough assessment using a parametric method has concluded that Fig. 6(B) shows a significant change in sensor response, and this relates to the placed HRCSRIB structure cobalt (Co)-based resonator length CoR_L_, becoming an exceptional sensitivity with 1750 nm/RIU for 342 mg/dL glucose level, 1666.66 nm/RIU for 205–289 mg/dL glucose level, and 1500 nm/RIU for 182 mg/dL glucose level. The higher rates of transmission with the values of 82%, 81%, 55%, 80% obtained in Fig. 6(A). The response of length dimensions CoR_L_ = 60 nm, and CoR_L_ = 70 nm has been showcasing in terms of lines of curved lines with no response of detecting peaks for glucose in Fig. 6(C) & Fig. 6(D). Figure 6(E) having two groups of high transmission rates glucose monitoring peaks with 75%, 3%, 75%, 77%, and 67%, 75%, 53%, 64%.

**Fig. 7:**
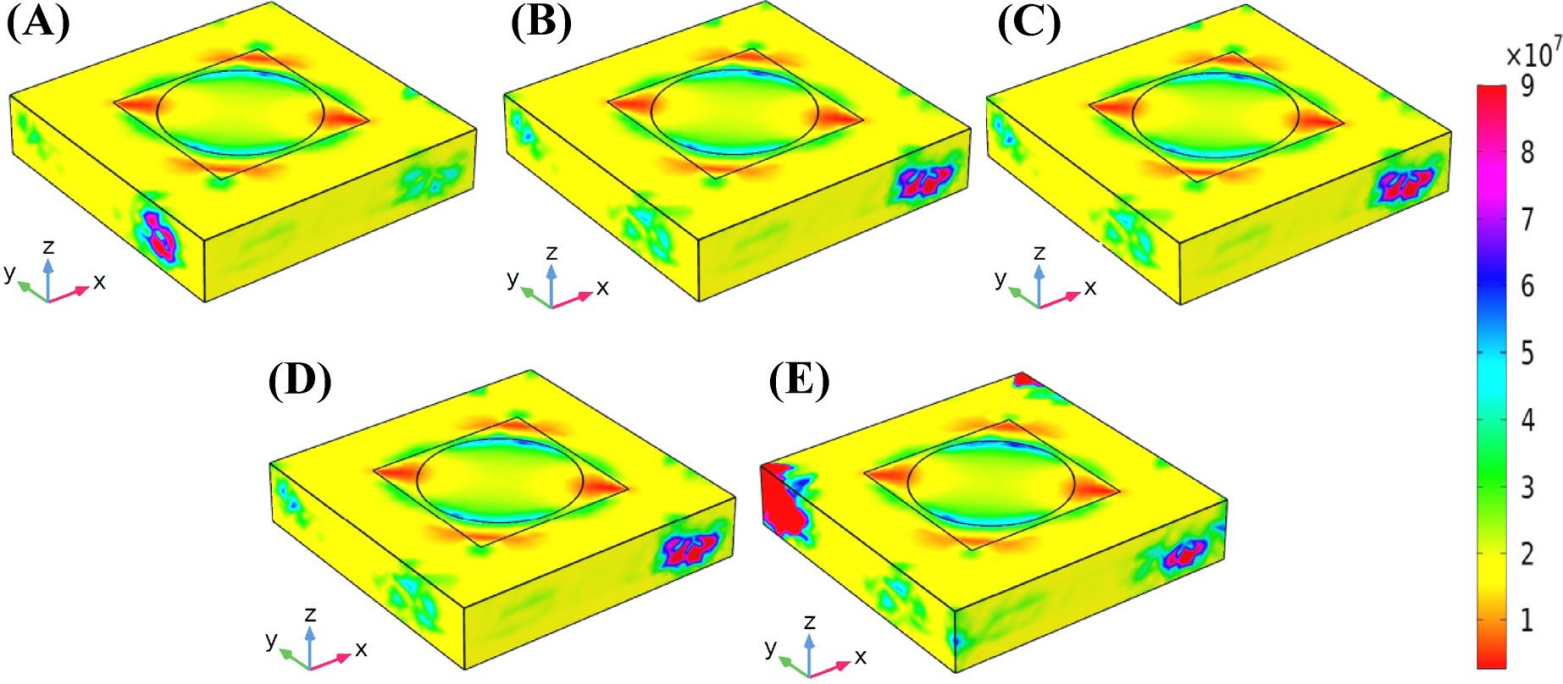
The screening of Electric Field Distribution (EFD) of the Hollow-Ring Cubic-Structured Refractive Index Biosensor (HRCSRIB).

The screening of the electric field distribution (three-dimensional distribution over the designed structure) of the Hollow-Ring Cubic-Structured Refractive Index Biosensor (HRCSRIB) is showcased in Fig. 7 with an indicator. Figures 7(A) and 7(E) showcase the EFD at the wavelengths of 2200 nm and 2400 nm, which indicates a normal wavelength. Figure 7(B) showcases the EFD at the wavelength of 2288 nm with a refractive index value of 1.337, which indicates a 182 mg/dL glucose level wavelength, while Fig. 7(C) showcases the EFD at the wavelength of 2290 nm with a refractive index value of 1.338, which indicates a 205, 220, 240, 255, 289 (mg/dL) glucose level wavelength, and Fig. 7(D) showcases the EFD at the wavelength of 2292 nm with a refractive index value of 1.339, which indicates a 342 mg/dL glucose level wavelength. This presented HRCSRIB with the EFD shows a progressive shift and intensified field concentration, as variation observed in wavelength and associated refractive index. The electric field gets stronger and more focused in the sensing regions as the wavelength increases from 2200 nm to 2292 nm, which corresponds to glucose levels from normal to elevated levels. The EFD at 2288 nm (RI = 1.337) showcases intensified confinement along the inner edges of the hollow ring. At 2290 nm (RI = 1.338), this confinement gets even more concentrated, which indicates significant light-matter interaction. At 2292 nm (RI = 1.339), the field demonstrates maximum localization, specifically at structural contacts, which shows the sensor’s response to small RI variations. These spatial field changes illustrate the HRCSRIB’s sensitivity as well as its ability to recognize small variations in glucose levels.

**Fig. 8:**
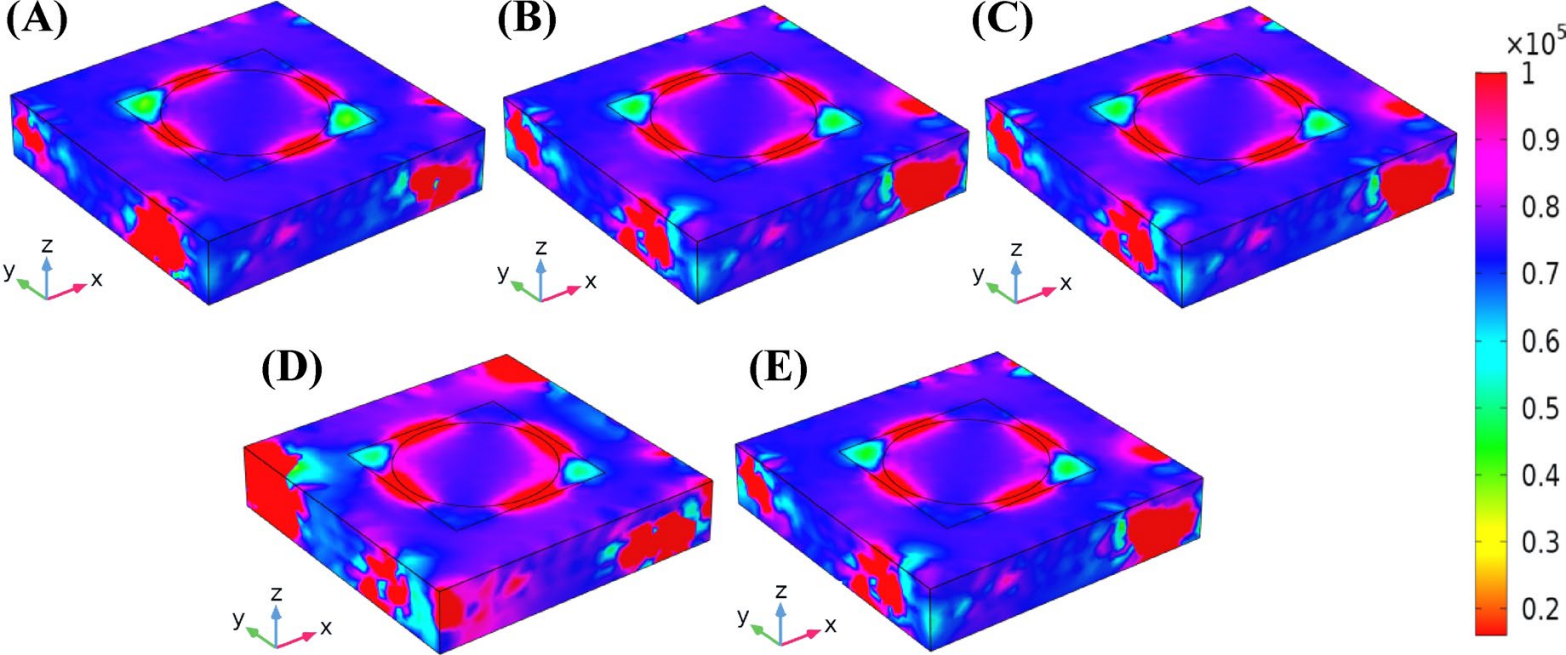
The screening of Magnetic Field Distribution (MFD) of the Hollow-Ring Cubic-Structured Refractive Index Biosensor (HRCSRIB).

The screening of the magnetic field distribution (three-dimensional distribution over the designed structure) of the Hollow-Ring Cubic-Structured Refractive Index Biosensor (HRCSRIB) is showcased in Fig. 8 with an indicator. Figures 8(A) and 8(E) showcase the MFD at the wavelengths of 2200 nm and 2400 nm, similar to the electric field, which indicates a normal wavelength. Figures 8(B), 8(C), and 8(D) showcase the MFD at the same wavelength and refractive index value as similar to the electric field distribution. This presented HRCSRIB with the MFD, Fig. 8(A) & 8(E) showcases modest magnetic field intensity at λ = 2200, and λ = 2400 (*n* = 1.335, normal glucose level), and Fig. 8(B) begins to showcase localized magnetic field confinement at λ = 2288 near the hollow-ring boundary (*n* = 1.337, 182 mg/dL glucose level). Further, Fig. 8(C) shows a narrowed and intensified magnetic field, which is concentrated around structural interaction areas. This would indicate enhanced light-matter interaction and a resonance shift response, which demonstrates the ability of the sensor to detect small RI variations (*n* = 1.338, 205–289 mg/dL glucose level). Figure 8(D) displays maximized localization of the magnetic field and intensity of the peak field. The resonating effect has been effectively determined, and the magnetic field distribution is strongly focused, which shows the HRCSRIB’s significant detection accuracy for higher glucose levels (*n* = 1.339, 342 mg/dL glucose level).

**Table 2:** Substantial parameters to measure a glucose concentration in the blood of Hollow-Ring Cubic-Structured refractive index biosensor (HRCSRIB).

Different concentrations of glucose (mg/dL) in blood sample	Refractive index variation of urine (*n*)	Difference in the refractive index (Δn)	Peak Wavelength (nm)	Sensitivity (nm/RIU)	Figure of merit (FOM)	Detection limit (DL)	Quality factor (QF)	Detection Range (DR)	Sensor Resolution (SR)	Signal Noise Ratio (SNR)
165–180	1.335	Ref.	2285	Ref.	1056.33	Ref.	1609.15	1920.16	Ref.	Ref.
182	1.337	0.002	2288	1500.00	1216.54	0.000488	1670.07	1955.55	0.7320	2.1897
205, 220, 240, 255, 289	1.338	0.003	2290	1666.66	1241.13	0.000410	1624.11	1940.67	0.6833	3.5460
342	1.339	0.004	2292	1750.00	921.05	0.000509	1206.31	1672.99	0.8907	3.6842

**Table 3:** The comparability of the offered Hollow-Ring Cubic-Structured refractive index biosensor (HRCSRIB) and existing biosensors.

Existing Biosensor	Sensitivity (nm/RIU)	Quality factor (QF)	Figure of merit (FOM)	Detection Limit (DL)
^31^2024, Jayakrishnan Kulanthaivel et al.	831.32	152.52	88.91	10^−3^
^32^2024, Esmat Rafiee et al.	1607	171.71	172.8	6.53 × 10^−4^
^33^2022, Fariborz Parandin et al.	2.94	5166	9.84	0.021
^34^2024, Hadi Sharifi et al.	900	190	105	0.00001
^35^2017, Farhad Mehdizadeh et al.	306.25	5000	103	-
^36^2019, Masoud Mohammad et al.	278.5	3382	-	0.0015
^37^2023, Bhuvaneshwari Krishnamoorthi et al.	166	3702	-	-
^38^2018, Fariborz Parandin et al.	546.72	2066.44	-	1.44
^39^2023, Abdelkader Abderrahmane et al.	832	10^5^	1.46 × 10^5^	3.4 × 10^−7^
^40^2023, Abdelkarim El Mouncharih et al.	965	1892.54	756.86	-
^41^2019, Rajendran Arunkumar et al.	-	262	-	0.002
**Proposed work**	**1750**	**1670.07**	**1241.13**	**0.000410**

The substantial Parameters along with all the micro-analysis research to measure glucose concentration in the blood of Hollow-Ring Cubic-Structured Refractive Index Biosensor (HRCSRIB) which is showcased in Table 2. The comparability of the offered Hollow-Ring Cubic-Structured Refractive Index Biosensor (HRCSRIB) with detailed expression and existing biosensors is showcased in Table 3. When compared with the current designs, the comparative data in Table 3 thoroughly demonstrate the higher performance of the proposed HRCSRIB. The HRCSRIB’s noteworthy sensitivity of 1750 nm/RIU, which substantially surpasses several reported values, such as 831.32 nm/RIU in^31^1607 nm/RIU in^32^2.94 nm/RIU in^33^900 nm/RIU in^34^546.72 nm/RIU in^38^832 nm/RIU in^39^965 nm/RIU in^40^and many more, highlights its remarkable ability to determine even the smallest variations in refractive index. Although some of the sensor design reports a bit higher quality factor, the offered biosensor still features a high QF of 1670.07, which indicates a well-balanced resonance sharpness suitable for everyday use. Additionally, its figure of merit (FOM) is 1241.13, which shows a desirable combination of high sensitivity and a narrow spectrum width, that is essential properties for accurate biosensing. Furthermore, with a detection limit of 0.000410, the HRCSRIB exhibits the capacity to recognize very low analyte concentrations, which makes it perfect for applications that require superior sensitivity and precision. Overall, the combination of the above performance metrics qualifies the HRCSRIB as a highly competitive and efficient device in the field of advanced refractive index biosensors.

## Conclusion

In summary, we have offered Hollow-Ring Cubic-Structured Refractive Index Biosensor (HRCSRIB) for monitoring glucose levels in blood from normal concentrations of 165 to 180 (mg/dL) in compared to diabetic glucose concentrations of 182, 205, 220, 240, 255, 289, 255 (mg/dL). As a result, employing cobalt as a resonator metal provides affordable pricing with a desirable substitute for more costly expensive metals such as silver and gold, thus enabling the development of highly effective and inexpensive biosensors. This offered biosensor has an exceptional sensitivity with 1750 nm/RIU for 342 mg/dL glucose level, 1666.66 nm/RIU for 205–289 mg/dL glucose level, and 1500 nm/RIU for 182 mg/dL glucose level which intensifies the transmission. The exceptional value of the quality factor, as well as the figure of merit value, is 1670.07 nm/RIU of 182 mg/dL and 1241.13 for 205–289 mg/dL. The exceptional value of detection limit with the 0.000410 and detection range of 1955.55 has been noted for 182 mg/dL concentration of glucose. The earlier mentioned parameters correlate effectively, making it possible for accurate measurement of the levels of glucose in the blood.

## Data Availability

The data used to support the findings of this study are included in the article. Data may be provided upon request from Shobhit K. Patel, Email: shobhitkumar.patel@marwadieducation.edu.in.
